# Case report: anaesthetic and surgical management of a diaphragmatic rupture with tension pneumothorax and iatrogenic bowel perforation in an undiagnosed Bochdalek hernia patient

**DOI:** 10.1186/s12871-022-01736-z

**Published:** 2022-06-24

**Authors:** Steffi Kang Ting Chan, Daryl Jian’an Tan, Maria Dhahrani Martinez Aman

**Affiliations:** Division of Anaesthesiology and Perioperative Medicine, Singapore General Hospital, Block 5 Level 2, Outram Road, Singapore, 169608 Singapore

**Keywords:** Diaphragmatic hernia, Emergency surgery, Abdominal surgery, Critical care, Airway management

## Abstract

**Background:**

Congenital diaphragmatic defects are rare, with most cases presenting in childhood. Diagnosis in adulthood is usually incidental or when symptoms develop. We present a case of a strangulated Bochdalek hernia complicated by possible tension pneumothorax and iatrogenic bowel injury in a healthy young male.

**Case presentation:**

A 23-year-old Chinese man initially presented with complaints of mild back pain and was discharged with symptomatic treatment. He presented again 3 days later, with dyspnea and left upper back pain and was haemodynamically unstable and hypoxic. A chest x-ray was reported as a moderately large left-sided pneumothorax with herniation of bowel into the left hemithorax. Needle decompression resulted in feculent fluid being aspirated with no resolution of symptoms. The patient required an immediate transfer to the operating theatre for surgical intervention of his left diaphragmatic rupture, complicated by visceral herniation and left tension pneumothorax, with accidental puncture of the herniated bowel. He underwent an emergent laparotomy with requirements for rapid lung isolation and continued aggressive resuscitation.

**Conclusions:**

Patients with congenital diaphragmatic hernias may present in adulthood, either incidentally or emergently. In the well adult patient with good reserves, these initial symptoms may be mild, and may be symptomatically treated with no further workup. However, patients may deteriorate rapidly once their compensatory mechanisms are exhausted.

This is the first reported case of a patient with diaphragmatic rupture and bowel herniation, complicated by iatrogenic tension pneumothorax. This rare case illustrates the speed at which a diaphragmatic rupture may progress, possible pitfalls and offers insights on how a misdiagnosis may be avoided.

## Background

Congenital diaphragmatic defects are rare, with the most defects being Bochdalek hernias that are defects in the posterolateral diaphragm allowing intra-abdominal contents to herniate into the thoracic cavity [1, 2]. While most cases are detected in the paediatric population, a few of them can remain undetected until adulthood with a reported incidence of 0.17% [3–6]. We present a case of a strangulated Bochdalek hernia complicated by tension pneumothorax and iatrogenic bowel perforation in a young male patient.

## Case presentation

A 23-year-old man of Chinese descent presented to the emergency department for dyspnoea and pain in the left upper back. He had initially presented to the emergency department after an episode of severe vomiting following alcohol consumption and mild back pain three days prior, and was treated symptomatically and discharged. The patient denied any recent trauma. He had no other past medical issues nor known congenital conditions.

Upon review, the patient was in severe respiratory distress and haemodynamically unstable with blood pressure of 95/70 mmHg, heart rate 162 beats per minute, respiratory rate of 40 breaths per minute and peripheral capillary oxygen saturation (SpO2) of 87% on 100% oxygen administered via non-rebreather mask. Although unable to lie still, he was oriented and able to speak in short phrases. His trachea was deviated and lung sounds were absent over his left chest.

**Fig. 1:**
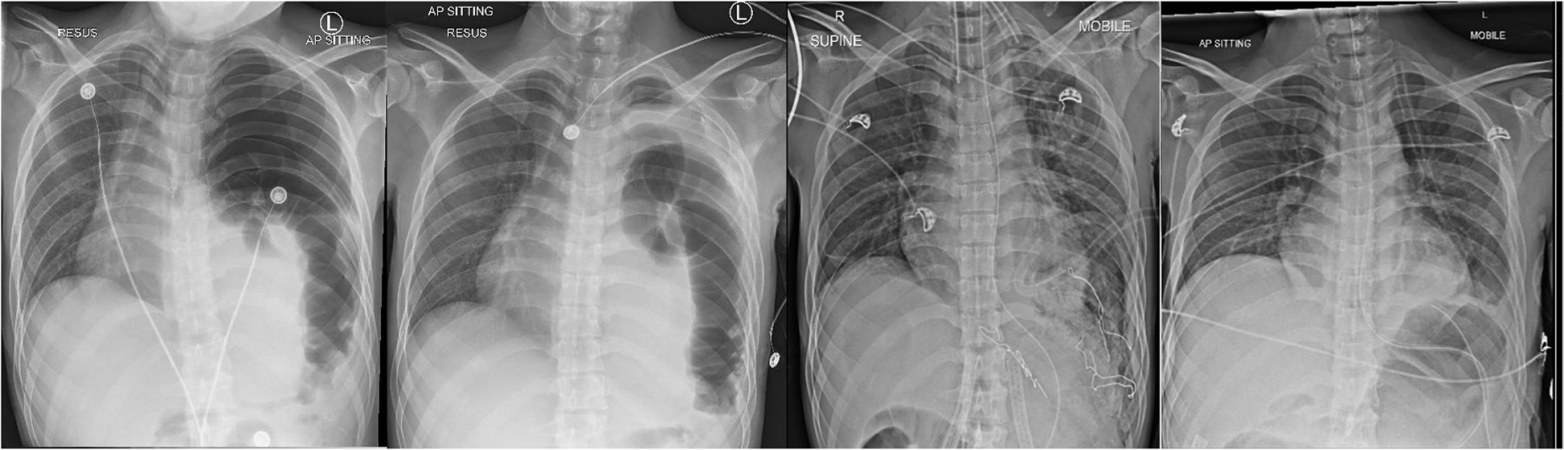
Chest X-ray images. **a** On admission showing a large left sided pneumothorax with tracheal deviation and herniation of bowel into the left hemithorax. **b** After attempted needle decompression showing a persistent left pneumothorax, with further elevation of the bowel loop superiorly in the left hemithorax. The mediastinum is slightly less deviated but still shifted to the right. **c** After the first surgery showing reduction of left diaphragmatic hernia and temporary closure of defect with surgical towels, There was also re-expansion of the left lung with airspace opacities in the mid and lower zones. **d** After discharge from ICU showing improvement of airspace opacities

A chest X-ray (CXR) performed was interpreted as a moderately large left-sided pneumothorax with herniation of bowel into the left hemithorax (Fig. 1a). Due to patient instability, no computer tomography (CT) scan was performed after the CXR. A 14-gauge intravenous cannula was inserted into the second intercostal space along the mid-clavicular line for needle decompression in the sitting position.. However, air, followed by approximately 900 ml of faecal fluid, was aspirated, with no improvement in the patient’s symptoms. A repeat CXR showed a slight improvement of tracheal deviation, but with further bowel herniation taking up most of the left lung field. There was still concern that the pneumothorax had not been completely relieved.

Arterial blood gas (ABG) indicated severe combined respiratory and metabolic acidosis (pH 7.212, PaCO2 34.6 mmHg, PaO2 282 mmHg, base excess -14 and serum bicarbonate 13.9 mmol L^-1^. Serum lactate levels were elevated at 3.2 mg dL^-1^).

The patient was immediately transferred to the operating theatre for surgical intervention of his left diaphragmatic rupture, complicated by visceral herniation and possible left tension pneumothorax, with iatrogenic puncture of the herniated bowel. After discussion with the surgeons, a repeated needle decompression or chest tube insertion prior to induction of anaesthesia was not attempted due to the high failure rate attributed to the herniated bowel’s position, uncertain anatomy and the patient’s inability to cooperate.

After adequate preoxygenation, rapid sequence induction was performed with cricoid pressure and intravenous propofol 150 mg, rocuronium 50 mg, and remifentanil target-controlled infusion at 1 ng ml^-1^. The SpO2 before induction was 92%, and the lowest SpO2 obtained while the airway was being secured was 80%. A left-sided double-lumen tube (DLT) was inserted and both the tracheal and bronchial cuffs were inflated immediately prior to the commencement of one lung ventilation. Rapid deflation of the left lung using a suction catheter was then carried out. Position of the left bronchial cuff was finally confirmed to be in the left main bronchus with a fiberoptic scope. Although the SpO2 improved to 100%, there was significant hypotension post-induction to lowest 63/45 mmHg, which was quickly addressed with boluses of phenylephrine and adrenaline to a total of 1000 μg and 30 μg respectively. An ABG obtained after induction showed pH 7.091, PaCO2 61.5 mmHg, PaO2 194 mmHg, base excess -11 and serum bicarbonate 18.7 mmol L^-1^.

Intraoperatively, a 10 cm by 6 cm Bochdalek diaphragmatic hernia defect with herniation of the greater curve of stomach, spleen and long loop of transverse colon into the left hemithorax was found. A large perforation with necrotic edges in the greater curve of the stomach was also noted, with feculent material in the pleural and peritoneal cavity. No other obvious defects of the small and large bowels were seen.

Simultaneous assessment and resuscitation of his persistent hypotension, tachycardia and hypoxia was carried out while surgery was ongoing. Based on the patient’s clinical history and operative findings, it was likely that he had a combination of obstructive, hypovolemic and distributive shocks due to tension pneumothorax, intravascular depletion and feculent peritonitis. He was aggressively fluid resuscitated with 1.8 litres of intravenous crystalloids and 2 litres of colloids. Noradrenaline and adrenaline infusions were commenced shortly after induction and maintained on 0.2 μg kg^-1^ min^-1^ for both infusions. His antibiotics were escalated from intravenous (IV) ceftriaxone and metronidazole to IV piperacillin-tazocin, and additional fungal coverage with IV anidulafungin was given. IV hydrocortisone was also administered. At the end of the surgery, he remained on noradrenaline 0.2 μg kg^-1^ min^-1^ and vasopressin 0.03 units kg^-1^ hr^-1^.

As the tension pneumothorax could not be surgically relieved until the herniated abdominal structures had been reduced, attempts were made to decompress the left pleural cavity with continued suction via the bronchial lumen of the left-sided DLT, to limited success. Eventually, a wedge gastrectomy of the stomach, diaphragmatic hernia reduction and left chest tube insertion was performed, followed by a temporary abdominal closure with the diaphragmatic defect being reinforced with surgical towels. Estimated blood loss was 800 ml and surgery lasted approximately 3 hours.

A repeat ABG at the conclusion of the surgery showed persistent combined respiratory and metabolic acidosis (pH 7.153, PaCO2 54.5 mmHg, PaO2 128 mmHg, base excess -10 and serum bicarbonate 19.1 mmol L^-1^). The double-lumen tube was exchanged for a single-lumen tracheal tube and the patient was sent to the Intensive Care Unit (ICU) for continued resuscitation. Postoperatively, a repeated CXR showed reduction of the left diaphragmatic hernia with re-expansion of the left lung (Fig. 1c). Dual vasopressor support was weaned off on Day 2 of admission and the patient did not have high ventilatory requirements.

The patient returned to the operating theatre for a relook surgery on Day 3 of admission. The hernia defect was repaired with a synthetic mesh and primary abdominal closure was performed uneventfully. The patient was extubated on Day 5 of admission and discharged from the ICU on Day 6 of admission. CXR on Day 6 of admission showed resolution of pneumothorax and reduction of diaphragmatic hernia (Fig. 1d). After an additional 2 weeks’ stay for total parenteral nutrition and post-operative recovery, he was discharged home 22 days after admission.

## Discussion and conclusions

Congenital diaphragmatic hernias (CDH) are idiopathic malformations of the diaphragm, which are usually present in the newborn period. Despite occurring in one to five babies per 1000 births, they are usually diagnosed early and are relatively uncommon in adulthood. Associated malformations are frequent and other organs may be affected. Bochdalek hernias are a subset of CDH which are characterised by a congenital defect on the posterolateral region of the diaphragm, without a hernia sac. It may be associated with other congenital abnormalities in up to 57% of cases and up to 20% of cases may have chromosomal disorders [3–6].

While infants with CDH may present with severely compromised respiratory and cardiovascular functions, adults may have a less drastic clinical presentation. The most frequent symptom in adults is mild discomfort and 25% of patients may not have any symptoms.

### Diagnosis of Bochdalek hernias

A chest radiograph is usually the mode of diagnosis, either as an incidental finding or in patients presenting with respiratory symptoms. The classical findings of CDH include intrathoracic gas-filled bowel loops with a contralateral shift of the mediastinum [7]. An air-fluid level may also be present. In late-presenting cases of CDH, it may be helpful to identify the gastric bubble position [8].

A computed tomography (CT) scan is the gold standard [9], providing an accurate assessment of the patient’s anatomy. CT scan has been shown to have sensitivity of 78% for left-sided hernias and 50% for right-sided hernias [9, 10]. The stomach, loops of small intestine or other components of the gastrointestinal tract may be seen in the hemithorax, herniating through the diaphragmatic defect. Patient-specific anatomical variations may be imaged, allowing for precise evaluation of the relationships between the abnormally-positioned viscera. Upper gastrointestinal and bowel double-contrast studies may also aid in further confirmation [7].

### Clinical challenges

The radiological features of CDH form a spectrum and may not always be easy to detect, even mimicking other pathologies. The absence of lung markings may result in it being mistaken for a pneumothorax. In other literature, it has also been mentioned that the bowel loops may appear as an opacity, similar to lung consolidations in pneumonia [11, 12], a massive pleural effusion [13] or even pericardial fat pads, mediastinal lipomas or an anterior mediastinal mass [14]. Hence, a careful analysis of chest films is required to avoid misdiagnosis. Although a CT scan allows for the highest accuracy of a correct diagnosis, it may be difficult to arrange and perform in a patient presenting in extremis [9–11].

In our patient, the chest x-ray showed bowel herniation with no obvious outline of the diaphragm. Although the team was aware of the presence of herniated intestinal loops, they were certain that there was a concurrent pneumothorax. However, given that the attempt at needle decompression also aspirated a large amount of feculent fluid, this may have been a misdiagnosis. The pneumothorax could have been a large loop of obstructed bowel. The improvement in mediastinal shift (Fig. 1b) was possibly due to the release of nearly 1 litre of intestinal contents via the cannula.

### Avoiding misdiagnosis and alternative modalities

Apart from having a high index of suspicion whenever the diaphragm cannot be adequately visualised on CXR and stabilising the patient enough to perform a quick CT scan (through the judicious use of sedatives and provision of supplemental oxygen as required), other ways to avoid this mistake would include a thorough clinical examination and the use of modalities such as a portable ultrasound.

In the critically ill patient, both tension pneumothorax and diaphragmatic hernia may present with hypoxia and hypotension. However, there are some differences in the clinical presentation which may raise suspicion for a congenital diaphragmatic hernia.

Patients with tension pneumothorax typically present with respiratory symptoms including chest pain (52.3%), dyspnea (38.4%) and shortness of breath (27%). Respiratory distress and tachypnea (respiratory rate greater than 20) are common, as well as the need for supplemental oxygen and a decreased PaO2/FiO2 ratio. Examination may reveal decreased air entry, percussion hyperresonance and decreased thoracic excursions. In the same systematic review, gastrointestinal symptoms were not reported [15].

In contrast, an acute presentation of diaphragmatic hernia in adulthood is usually due to incarceration, obstruction, or strangulation of the herniated viscera [7]. Hence, abdominal symptoms may dominate and a history of chest discomfort and abdominal pain (81%), breathlessness (61.9%) and vomiting (47.6%) may point towards herniation of intra-abdominal viscera. Auscultation of bowel sounds in the chest and paradoxical movements of the abdomen may also be seen [16].

Although uncommon, ultrasound may also be used to differentiate both conditions in an emergent setting. Pneumothorax typically presents with a lack of lung sliding, absence of B lines and a “barcode sign” on M mode. The junction between sliding lung and absent lung sliding (lung point sign) is 100% specific for pneumothorax [17]. Features to look out in diaphragmatic rupture and bowel hernia include fluid collection in the pleural or subphrenic spaces, a disrupted appearance of the diaphragm, as well as visualisation of bowel peristalsis and small bowel mucosa folds. Hepatic or splenic parenchymal movement may also be present against the parietal pleural surface when lung parenchyma is normally seen [18]. However, this is limited by operator unfamiliarity and the patient’s ability to cooperate.

### Lung isolation techniques in an emergent setting

Immediate lung isolation was necessary in this case, as it was unclear if a tension pneumothorax was present. Even if there were no pneumothorax initially, the attempted needle aspiration could have resulted in one. Positive pressure ventilation would have been detrimental in the case of pleural disruption. Thus, there was a need to avoid ventilating the affected hemithorax while surgical exploration was ongoing.

Lung isolation involves utilising bronchial barrier devices to allow each lung to function as a separate unit. In this patient, there was a need for emergent isolation of the affected lung, to avoid further airway compromise and impairment to ventilation. The usual airway management strategies for emergency one-lung ventilation have been well-described and include the use of a DLT, endotracheal tube with bronchial blocker (BB), or intentional endobronchial intubation [19].

DLT is the most widely-used device for ensuring effective lung isolation. Its bifurcated structure allows for independent ventilation of the tracheal or endobronchial lumens. A suction catheter can also be passed down either lumen to facilitate lung deflation if surgically indicated. This is particularly pertinent in our case, where the presence of an untreated tension pneumothorax and diaphragmatic rupture have already resulted in hemodynamic instability, and there is an urgency for lung isolation to prevent progression of the patient’s obstructive shock. However, owing to its more bulky profile, the DLT is more challenging to insert, with increased risks of dental, oropharyngeal and laryngeal injuries. Loss of airway during attempts to insert DLT would contribute to hypoxia in an already compromised patient. Our patient was also at a high risk of aspiration, with a need to secure the airway as soon as possible. Furthermore, in this patient, who was planned for a transfer to the intensive care unit after surgery, there would also need to be an exchange of the patient’s airway to a regular endotracheal tube to facilitate care in the ICU and avoid to iatrogenic airway trauma from prolonged ventilation with a DLT.

BBs are an alternative to achieve lung isolation. By passing a BB down an endotracheal tube to occlude the main stem bronchus, ventilation distal to the occlusion may be prevented. There is no need for a tube exchange at the end of surgery as the BB can simply be removed with the endotracheal tube left in situ. However, more time may be needed to achieve effective lung isolation, as all BBs need to be positioned under fiberoptic bronchoscopic guidance. It is also not as effective in facilitating deflation of the compromised lung, as there is no separate channel available for gas to escape through.

The last option would be to use a single lumen tube, advancing it into the main bronchus of the non-operative lung. In a majority of patients, the right main bronchus has a trajectory that approximates that of the trachea, and would be selectively intubated. As our patient’s pathology was in his left lung, lung isolation with this technique would be viable. However, bronchoscopy, suctioning would not be possible in the isolated lung.

In deciding on our choice of airway, our priorities were 1) securing an airway 2) immediate lung isolation.

Although our patient’s agitation precluded a detailed airway assessment, he had no significant features of a difficult airway. Hence, we were confident of our ability to insert a DLT and for tube exchange at the end of surgery.

Assuming that it was in the correction position, the DLT would also allow us to isolate the lung immediately after intubation and before commencing positive pressure ventilation, as its position could be checked without the need for a bronchoscope, which was a significant advantage over using a bronchial blocker. As the diagnosis of tension pneumothorax was unclear at this point in time, we felt that this was ideal to avoid exacerbating any potential intrapleural pathology.

## Conclusion

CDHs are usually present in childhood, but may lie undiagnosed till adulthood. Patients with congenital diaphragmatic hernias may present in adulthood, either incidentally or emergently. In the well adult patient with good reserves, these initial symptoms may be mild, and may be symptomatically treated with no further workup. However, patients may deteriorate rapidly once their compensatory mechanisms are exhausted.

Multiple issues have to be addressed during simultaneous diagnosis, resuscitation and management. Vigilance is required to be able to accurately identify CDHs in the adult presenting in extremis, in order to avoid misdiagnosis and mismanagement.

Lastly, a good perspective of the issues and good communication between members of the anaesthetic, surgical and intensive care teams is crucial to ensure effective care in a high-stakes scenario.

## Data Availability

Data sharing is not applicable to this article as no datasets were generated or analysed during the current study.

## Abbreviations

SpO2: peripheral capillary oxygen saturation
CXR: chest x-ray
ABG: arterial blood gas
DLT: double lumen tube
PaCO2: partial pressure of carbon dioxide in arterial blood
PaO2: partial pressure of oxygen in arterial blood
ICU: intensive care unit
CDH: congenital diaphragmatic hernia
CT scan: computed tomography scan
FiO2: fraction of inspired oxygen
BB: bronchial blocker
